# Clinical Features and Complications of *Coxiella burnetii* Infections From the French National Reference Center for Q Fever

**DOI:** 10.1001/jamanetworkopen.2018.1580

**Published:** 2018-08-24

**Authors:** Cléa Melenotte, Camélia Protopopescu, Matthieu Million, Sophie Edouard, M. Patrizia Carrieri, Carole Eldin, Emmanouil Angelakis, Félix Djossou, Nathalie Bardin, Pierre-Edouard Fournier, Jean-Louis Mège, Didier Raoult

**Affiliations:** 1 Aix-Marseille University, Institut de Recherche pour le Développement (IRD), Assistance Publique Hôpitaux de Marseille (APHM), Microbes, Evolution, Phylogénie et Infections, IHU (Institut Hospitalo-Universitaire)–Méditerranée Infection, Marseille, France; 2 French Reference Center for the Diagnosis and Study of Rickettsioses, Q Fever and Bartonelloses, IHU–Méditerranée Infection, Marseille, France; 3 Observatoire Régional de la Santé Provence-Alpes-Côte d’Azur, Marseille, France; 4 Aix-Marseille Université, Institut National de la Santé et de la Recherche Medicale, IRD, Sciences Economiques et Sociales de la Santé et Traitement de l’Information Médicale, Marseille, France; 5 Unité de Maladies Infectieuses et Tropicales, Centre Hospitalier André Rosemon, Cayenne, Guyane Française; 6 Immunology Laboratory, APHM, Centre Hospitalier Universitaire Conception, Marseille, France

## Abstract

**Importance:**

Q fever remains widespread throughout the world; the disease is serious and causes outbreaks and deaths when complications are not detected. The diagnosis of Q fever requires the demonstration of the presence of *Coxiella burnetii* and the identification of an organic lesion.

**Objective:**

To describe the hitherto neglected clinical characteristics of Q fever and identifying risk factors for complications and death.

**Design, Setting, and Participants:**

This prospective cohort study conducted from January 1, 1991, through December 31, 2016, included patients treated at the French National Reference Center for Q fever with serologic findings positive for *C burnetii* and clinical data consistent with *C burnetii* infection. Clinical data were prospectively collected by telephone. Patients with unavailable clinical data or an unidentified infectious focus were excluded.

**Main Outcomes and Measures:**

Q fever complications and mortality.

**Results:**

Of the 180 483 patients undergoing testing, 2918 had positive findings for *C burnetii* and 2434 (68.8% men) presented with clinical data consistent with a *C burnetii* infection. Mean (SD) age was 51.8 (17.4) years, and the ratio of men to women was 2.2. At the time of inclusion, 1806 patients presented with acute Q fever, including 138 with acute Q fever that progressed to persistent *C burnetii* infection, and 766 had persistent focalized *C burnetii* infection. Rare and hitherto neglected foci of infections included lymphadenitis (97 [4.0%]), acute Q fever endocarditis (50 [2.1%]), hemophagocytic syndrome (9 [0.4%]), and alithiasic cholecystitis (11 [0.4%]). Vascular infection (hazard ratio [HR], 3.1; 95% CI, 1.7-5.7; *P* < .001) and endocarditis (HR, 2.4; 95% CI, 1.1-5.1; *P* = .02) were associated with an increased risk of death. Independent indicators of lymphoma were lymphadenitis (HR, 77.4; 95% CI, 21.2-281.8; *P* < .001) and hemophagocytic syndrome (HR, 19.1; 95% CI, 3.4-108.6; *P* < .001). The presence of anticardiolipin antibodies during acute Q fever has been associated with several complications, including hepatitis, cholecystitis, endocarditis, thrombosis, hemophagocytic syndrome, meningitis, and progression to persistent endocarditis.

**Conclusions and Relevance:**

Previously neglected foci of *C burnetii *infection include the lymphatic system (ie, bone marrow, lymphadenitis) with a risk of lymphoma. Cardiovascular infections were the main fatal complications, highlighting the importance of routine screening for valvular heart disease and vascular anomalies during acute Q fever. Routine screening for anticardiolopin antibodies during acute Q fever can help prevent complications. Positron emission tomographic scanning could be proposed for all patients with suspected persistent focused infection to rapidly diagnose vascular and lymphatic infections associated with death and lymphoma, respectively.

## Key Points

**Question:**

What are the characteristics and clinical presentations of *Coxiella burnetii* infection using 21st-century–clarified definitions?

**Finding:**

In a cohort study of 2434 patients with Q fever, the following new critical Q fever foci were identified: acute endocarditis, lymphadenitis, and bone marrow involvement in hemophagocytic syndrome. Lymphadenitis is a risk factor for lymphoma, and the elevation of IgG anticardiolipin antibody titers in acute Q fever is associated with complications.

**Meaning:**

Screening for anticardiolipin antibodies may help prevent acute Q fever complications; the use of transthoracic echocardiography in acute Q fever and positron emission tomographic scanning in suspected persistent focalized infection is justified to improve the care of patients with Q fever.

## Teaser

This cohort study describes the clinical characteristics and identifies risk factors for complications and death among a large sample of French patients with Q fever.

## Introduction

Q fever (or Query fever) is a globally widespread zoonosis that was first described in 1937. The previous dichotomy between acute and chronic Q fever, which was based on serologic criteria, has led to the current confusion between chronic infection and post–Q fever fatigue syndrome.^1^ The diagnosis of infection requires the demonstration of an organic lesion (identified as the infectious focus) and evidence of a microbial infection (as proven by serologic findings, polymerase chain reaction analysis, culture, and/or immunohistochemical analysis using anti–*Coxiella burnetii* antibodies).^2,3,4,5^ This concept of the disease and paradigm shift was made possible by revolutionary improvements in imaging during the 21st century. The systematic and early use of transthoracic echocardiography (TTE) and positron emission tomographic (PET) scanning has allowed the identification of infectious foci that were previously undetected and that are now crucial and decisive for the diagnosis of *C burnetii* infection and to guide the choice of therapeutic approach. Therefore, new definition criteria have recently been published for *C burnetii* endocarditis, vascular infection, osteoarticular infection, lymphadenitis, and interstitial lung disease.^2,3,4,5^ Consequently, the term *chronic Q fever* should no longer be used.^2^

This change is particularly important because the serologic response is strain dependent. As an example, IgG antibody titers to phase I *C burnetii* are higher in French Guiana, where a unique strain is endemic, than in metropolitan France.^6,7^ For this reason, Q fever postinfectious syndrome is defined by an association of elevated IgG titers to phase I *C burnetii* with subjective symptoms only. This definition is different from that of the Netherlands team, who consider these cases to be chronic infection.^1^ The 26-year experience of the French National Reference Center for Q fever, which contains data collected prospectively from patients worldwide, gave us the opportunity to reanalyze *C burnetii* infection using the clarified 21st century definition and to highlight new, rare, and unusual infectious foci of the disease.

## Methods

### Study Design and Setting

The French National Reference Center for Q fever is designed by the French government to collect data from patients with Q fever as part of epidemiologic surveillance and receives serum samples from France and abroad (eFigure 1 in the Supplement). Epidemiologic, clinical, and biological data from positive cases are collected as described later. In this prospective cohort study, we report the epidemiologic, clinical, and biological data collected in the French National Reference Center from January 1, 1991, to December 31, 2016. We followed the Strengthening the Reporting of Observational Studies in Epidemiology (STROBE) reporting guidelines.^8^ According to the procedures of the French Commission for Data Protection (Commission Nationale de l’Informatique et des Libertés), collected data were anonymized. This study was exempt from ethical review and approval per Article L1121-4 of the French Public Health Code. An oral consent was obtained from study participants by the referral physician.

### Participants and Follow-up

For each positive *C burnetii* test finding, clinical data were collected by telephone from the referral physician using a standardized questionnaire (eFigure 2 in the Supplement). Patients with positive serologic test results who presented with clinical manifestations consistent with an active *C burnetii* infection were included. We asked the referral physician for serologic tests 3 and 6 months after diagnosis to monitor clinical and serologic markers of improvement. The duration of monitoring was set at 5 years in case of persistent focalized infections. Patients with unavailable clinical data or unidentified infectious focus were excluded from the study analysis.

### Diagnosis of *C burnetii* Infection

Serologic testing and molecular detection were performed as previously described.^9,10,11^ Culture and immunohistochemistry using specific anti–*C burnetii* antibodies and fluorescence in situ hybridization were performed as previously described.^9,12,13,14,15^

### Case Definition

Primary (acute) *C burnetii* infection was defined by the association of acute clinical symptoms with the following serologic criteria: IgG titers representing phase II (≥200) and IgM titers representing phase II (≥50) or seroconversion within 3 months of the primary symptoms.^16^ Persistent *C burnetii* focal infection was diagnosed using the recently updated criteria as persistence of clinical symptoms for more than 3 months in addition to the identification of an infectious focus (eTables 1-3 in the Supplement).^2,3,4^ Immunosuppression was defined in patients with known organ deficiency (those undergoing hemodialysis or before transplant), patients who were receiving an immunosuppressive drug or who underwent splenectomy, and patients with polymetastatic cancer.

### Biological Variables

Findings for G-isotype anticardiolipin (IgG aCL) antibodies were defined as positive at greater than 22 IgG anti–phospholipid-binding units (GPLU). Since 2012, tests have been systematically performed when an active *C burnetii* infection has been identified.^17,18^

### Imaging

For all patients with a positive *C burnetii* serologic test result since 2001, we recommend a cardiac TTE to detect known or unknown valvular defects or new valvular lesions compatible with endocarditis, because these conditions require prophylactic or curative treatment.^17,18^ Since 2009, the use of a PET scan is systematically recommended, when accessible, to detect deep infectious foci when focalized persistent infection is suspected.^2^

### Statistical Analysis

To compare the distribution of continuous or dichotomous variables between 2 groups, we used the 2-sided *t* test or the 2-sided Fisher test, respectively. Two-sided *P* < .05 was considered to indicate a significant difference between 2 groups. The mortality rate was computed as the number of deaths occurring in the cohort divided by the number of person-years during the study period. The Cox proportional hazards regression model was used to determine the factors associated with mortality risk. The proportional hazards assumption was tested in the Cox regression models by examining the rescaled Schoenfeld residuals. The indicative factors for complications were determined using the logistic regression model (for evolution to a persistent focalized *C burnetii* infection), the Poisson regression model (in cases of rare manifestations of acute Q fever), or the Cox proportional hazards regression model (for lymphoma). All multivariate models were adjusted for sex, age, and year category at baseline (before 2009, 2009-2012, and after 2012), and interactions were also tested. We used Stata/SE software (version 14.2; StataCorp LP) for all the analyses.

## Results

### Clinical Presentation of the Whole Cohort

**Figure 1.**
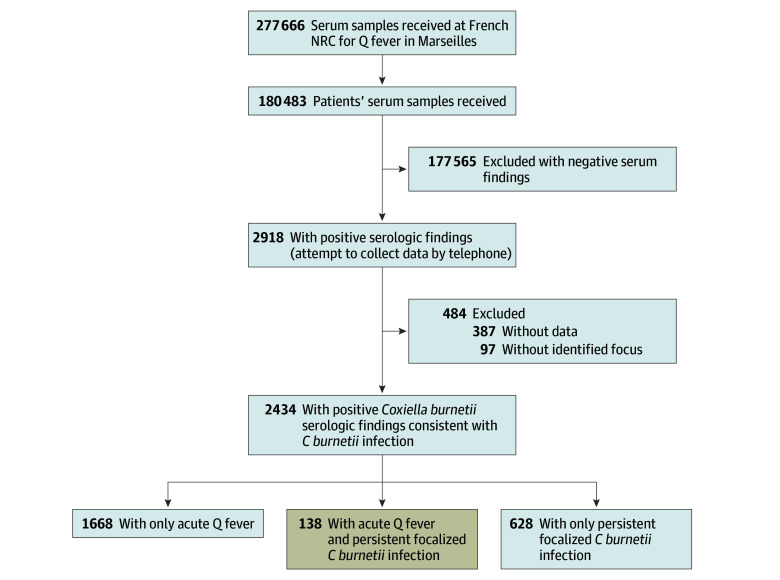
Study Flowchart Among the 2434 patients included in the study analysis, 1668 had only acute Q fever, 628 had only persistent focalized *C burnetii* infection, and 138 had an acute Q fever that evolved to a persistent *C burnetii* infection. NRC indicates National Reference Center.

From 1991 to 2016, 277 666 serum specimens were tested for antibodies to *C burnetii* (eFigure 1 in the Supplement). Of 180 483 patients undergoing testing, 2918 had positive findings for *C burnetti*. Of these, 2434 had a positive serologic result and an identified infectious focus (1674 [68.8%] men and 760 [31.2%] women) (Figure 1). A total of 2105 patients (86.5%) lived in metropolitan France, and 222 (9.1%) lived in Latin America, mostly in French Guiana (eTable 4 in the Supplement). The mean (SD) age of patients was 51.8 (17.4) years (range, 0-98 years); 58 (2.4% of the patients) were 16 years or younger. The ratio of men to women was 2.2 in adults and 0.9 in children (27 males [46%] and 31 females [54%]) (eFigure 3 in the Supplement). Mean (SD) follow-up was 16 (29) months. The medical records and cardiac TTEs at the time of diagnosis revealed that 640 patients (26.3%) presented with a valvulopathy, 91 (3.7%) were immunosuppressed (eTable 5 in the Supplement), and 36 (1.5%) were pregnant women (eTable 6 in the Supplement). Positron emission tomographic scanning was performed for 291 patients (12.0%) (eFigure 4 in the Supplement).

Among the 2434 patients included, 602 (24.7%) had a single-day follow-up. Nine hundred twenty-four of 1806 patients with acute Q fever (51.2%) were followed up for more than 3 months, and 149 of 766 patients with persistent *C burnetii* infection (19.5%) were followed up for more than 5 years.

### *C burnetii* Infection

**Figure 2.**
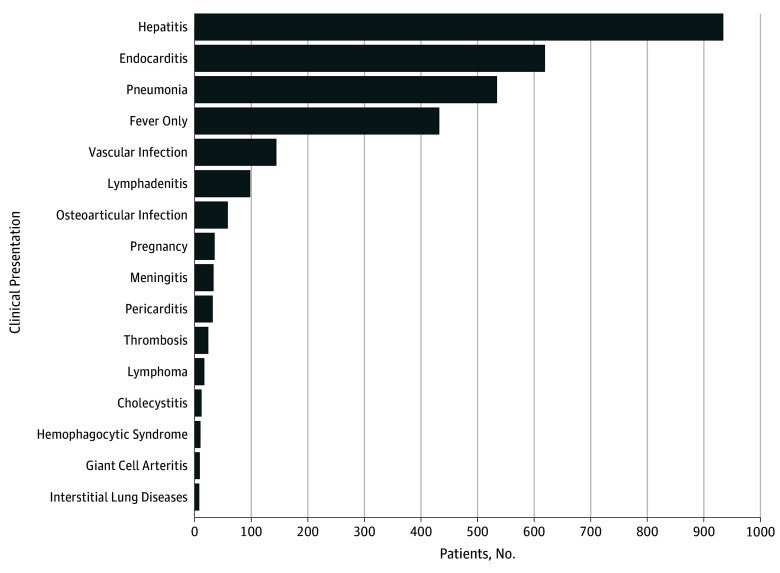
Clinical Presentations of *Coxiella burnetii* Infection Includes a total of 2434 patients with positive *C burnetti* serologic findings consistent with *C burnetti* infection.

In the overall cohort, 1806 patients (74.2%) had acute Q fever, and 31.5% (766 of 2434) presented with a persistent focalized infection. Among the 2434 patients, hepatitis was the most frequent clinical form of Q fever (933 [38.3%]), followed by endocarditis (533 [21.9%]), and pneumonia (618 [25.4%]) (Figure 2). Hepatitis (836 [46.3%]), pneumonia (480 [26.6%]), and flulike syndrome (350 [19.4%]) were the main clinical presentations of acute Q fever, followed by lymphadenitis (97 [5.4%]) (eTables 7 and 8 in the Supplement).

Among the 766 patients diagnosed as having persistent focalized *C burnetii* infection, 581 (75.8%) presented with endocarditis, 145 (18.9%) had a vascular infection, and 56 (7.3%) had an osteoarticular infection (eTables 9-11 in the Supplement). The mean (SD) age at diagnosis was 60 (17) years for these 766 patients, and the median duration of follow-up was 15.1 months (interquartile range, 1.7-45.3 months). In 91 patients (15.7% of patients with a final diagnosis of endocarditis), the use of transesophageal echography was critical to identify a valvular lesion, which was not detected with TTE. We witnessed the evolution of acute Q fever to a persistent *C burnetii* infection in 138 patients (7.6%) (Table 1).

**Table 1.** Evolution to Persistent *Coxiella burnetii* Infection in 1806 Patients With Acute Q Fever

Patient Characteristic	No. (%) of Patients		Univariate Analysis *P* Value^a^	Logistic Regression			
	Acute Q Fever Without Persistent *C burnetii* Infection (n = 1668)	Acute Q Fever Progressing to Persistent *C burnetii* Infection (n = 138)		Univariate		Multivariate	
				OR (95% CI)	*P* Value	OR (95% CI)	*P* Value
Immunosuppression							
No	1608 (96.4)	132 (95.7)	NA	1 [Reference]	NA	NA	NA
Yes	60 (3.6)	6 (4.3)	.63	1.2 (0.5-2.9)	.65	NR	NA
Valvulopathy							
No	1498 (89.8)	53 (38.4)	NA	1 [Reference]	NA	1 [Reference]	NA
Yes	170 (10.2)	85 (61.6)	<.001	14.1 (9.7-20.6)	<.001	9.8 (6.1-15.8)	<.001
Sex							
Male	1115 (66.8)	110 (79.7)	.002	1.9 (1.3-3.0)	.002	1.9 (1.1-3.1)	.01
Female	553 (33.2)	28 (20.3)	NA	1 [Reference]	NA	1 [Reference]	NA
Age at baseline, median (IQR), y	48 (37-59)	55.5 (46-68)	<.001	1.03 (1.02-1.04)	<.001	1.01 (1.00-1.03)	.03
Year category at baseline							
Before 2009	186 (11.2)	55 (39.9)	NA	1 [Reference]	NA	1 [Reference]	NA
2009-2012	680 (40.8)	32 (23.2)	NA	4.6 (3.1-7.0)	<.001	3.2 (1.9-5.3)	<.001
After 2012	802 (48.1)	51 (37.0)	.03	0.7 (0.5-1.2)	.19	0.8 (0.5-1.4)	.42
Pneumonia							
No	1211 (72.6)	115 (83.3)	NA	1 [Reference]	NA	1 [Reference]	NA
Yes	457 (27.4)	23 (16.7)	.005	0.5 (0.3-0.8)	.007	0.6 (0.3-1.0)	.07
Lymphadenitis							
No	1616 (96.9)	124 (89.9)	NA	1 [Reference]	NA	1 [Reference]	NA
Yes	52 (3.1)	14 (10.1)	<.001	3.5 (1.9-6.5)	<.001	3.3 (1.6-7.1)	.002
Thrombosis							
No	1657 (99.3)	133 (96.4)	NA	1 [Reference]	NA	1 [Reference]	NA
Yes	11 (0.7)	5 (3.6)	.005	5.7 (1.9-16.5)	.002	6.8 (1.9-24.8)	.004
Acute endocarditis							
No	1636 (98.1)	120 (87.0)	NA	1 [Reference]	NA	1 [Reference]	NA
Yes	32 (1.9)	18 (13.0)	<.001	7.7 (4.2-14.1)	.001	3.8 (1.5-9.8)	.006
IgG titer to phase I on first serologic analysis							
≤800	1476 (88.5)	84 (60.9)	NA	1 [Reference]	NA	NA	NA
>800	192 (11.5)	54 (39.1)	<.001	4.9 (3.4-7.2)	<.001	NR	NA
Maximum IgG titer to phase I							
≤800	1313 (78.8)	48 (34.8)	NA	1 [Reference]	NA	1 [Reference]	NA
>800	354 (21.2)	90 (65.2)	<.001	7.0 (4.8-10.1)	<.001	5.2 (3.3-8.1)	<.001
IgG aCL antibody titer							
≤90 GPLU	722 (77.4)	59 (67.8)	NA	1 [Reference]	NA	NA	NA
>90 GPLU	211 (22.6)	28 (32.2)	.048	1.6 (1.0-2.6)	.046	NR	NA
Positive *C burnetii* PCR							
No	1481 (91.3)	105 (77.8)	NA	1 [Reference]	NA	1 [Reference]	NA
Yes	142 (8.7)	30 (22.2)	<.001	3.0 (1.9-4.6)	<.001	1.9 (1.0-3.4)	.03

Abbreviations: GPLU, IgG anti–phospholipid-binding units; IgG aCL, G-isotype anticardiolipin; IQR, interquartile range; NA, not applicable; NR, not retained in the model; OR, odds ratio; PCR, polymerase chain reaction.

^a^ Calculated using the 2-sided Fisher exact test or 2-sided *t* test.

### New Clinical Presentations

#### Lymphadenitis and Lymphoma

Lymphadenitis was identified in 97 patients (4.0%). Lymphadenitis was concomitant with persistent focal *C burnetii* infection in 36 of 97 cases and was the unique infective focus in 23 cases (eTable 8 and eFigure 5 in the Supplement). Positron emission tomographic scanning enabled the identification of deep lymphadenitis in 18 of 41 cases (43.9%).

In patients diagnosed with Q fever, 16 were diagnosed as having a lymphoma. Fourteen (87.5%) were men, 14 had B-cell non–Hodgkin lymphoma, and 2 had T-cell lymphoma (6 diffuse large B-cell lymphomas, 2 follicular lymphomas, 1 gastric lymphoma, 1 mucosa-associated lymphoid tissue lymphoma, 2 marginal zone lymphomas, 1 mantle cell lymphoma, and 1 lymphoplasmacytic lymphoma) (eFigure 6 in the Supplement). Fourteen patients with lymphoma presented lymphadenitis. One patient had concomitant hemophagocytic syndrome and no lymphadenitis.

#### Coagulation Disorder, Elevated aCL Titers, and Acute Q Fever Endocarditis

Acute Q fever endocarditis (50 patients [2.1%]) was a risk factor for evolution to persistent focal *C burnetii* infection. Acute Q fever endocarditis occurred with hepatitis in 25 cases (50.0%) and with pneumonia in 13 (26.0%).

#### Thrombosis

Thrombosis was diagnosed in 23 patients of our cohort. Thrombosis was concomitant with persistent focalized *C burnetii* infection in 12 cases, including 8 with endocarditis, 5 with vascular infections, and 1 with osteoarticular infection (3 patients presented with endocarditis and vascular infection).

### Atypical Forms of Q Fever

Among the 2434 patients included in the analysis, neurologic involvement was diagnosed in 32 (1.3%). Fifteen patients presented with meningoencephalitis; 11, with meningitis; and 6, with encephalitis. In 25 of these patients, the neurologic infection occurred as a part of acute Q fever; in 7, it occurred as part of persistent endocarditis. Three patients who developed encephalitis as a complication of septic emboli died.

Thirty-one patients of the cohort (1.3%) had pericarditis. Twenty-three had acute Q fever, 11 had a persistent *C burnetii* infection, and 3 had both. Among the 7 patients with myocarditis, 2 developed severe complications, including 1 conduction defect and 1 case of endomyocardial fibrosis.

Eye involvement (n = 10) was the unique possible persistent focus of *C burnetii* infection in 7 cases and was concomitant with persistent lymphadenitis, pneumonia, and endocarditis in 1 patient each. Uveitis was the main observed manifestation (n = 7), followed by papillitis (n = 2), chorioretinitis (n = 1), and optic neuritis (n = 1). One patient presented with uveitis and papillitis.

Alithiasic cholecystitis was diagnosed in 11 patients (0.4%). In 4 of the 5 patients for whom IgG aCL was measured, these antibody titers were elevated (Table 2). For 2 patients, histologic analysis of the gallbladder showed inflammatory infiltrates, but immunohistochemical analysis yielded negative findings in both cases.

**Table 2.** Positive aCL Antibodies Associated With Clinical Complications of *Coxiella burnetii* Infection in 1328 Patients With Available IgG aCL Titers

Acute Q Fever Manifestation	No. (%) of Patients		Univariate Analysis *P* Value^a^	Multivariate Logistic Regression^b^	
	IgG aCL ≤22 GPLU (n = 830)	IgG aCL >22 GPLU (n = 498)		OR or IRR (95% CI)	*P* Value
Pneumonia (n = 319)	236 (28.4)	83 (16.7)	<.001	0.5 (0.4-0.6)^c^	<.001
Hepatitis (n = 503)	217 (26.1)	286 (57.4)	<.001	3.7 (2.9-4.7)^c^	<.001
Cholecystitis (n = 5)	1 (0.1)	4 (0.8)	.07	6.9 (0.7-62.8)^d^	.09
Hemophagocytic syndrome (n = 9)	0	9 (1.8)	<.001	NR	NR
Acute endocarditis (n = 42)	13 (1.6)	28 (5.6)	<.001	3.9 (2.0-7.5)^d^	<.001
Thrombosis (n = 21)	10 (1.2)	11 (2.2)	.18	2.1 (0.9-5.2)^d^	.09

Abbreviations: aCL, anticardiolipin; GPLU, IgG anti–phospholipid-binding units; IRR, incidence rate ratio; NR, not retained in this model; OR, odds ratio.

^a^ Calculated using the 2-sided Fisher exact test or χ^2^ test.

^b^ All multivariate models are adjusted for sex, age, and year category at baseline (before 2009, 2009-2012, and after 2012).

^c^ Odds ratio calculated using multivariate logistic regression.

^d^ Incidence rate ratio calculated using multivariate Poisson regression.

Hemophagocytic syndrome was diagnosed in 9 patients (0.4%), all with acute Q fever. In 1 patient, evolution to endocarditis occurred; 1 had evolution to vascular *C burnetii* infection; and 1 presented with a marginal B-cell lymphoma diagnosed based on a splenic biopsy. All 9 patients had elevated IgG aCL titers (>22 GPLU) (Table 2).

Seven patients with persistent focal *C burnetii* infection presented with interstitial lung disease. All patients had severe and advanced fibrotic lung lesions.^19^ A pseudotumor of the lung was detected in 3 patients. One had persistent endocarditis, 1 had persistent lymphadenitis, and 1 had acute Q fever.^18^

Seven patients with positive findings for *C burnetii* infection presented with giant cell arteritis, 5 had acute Q fever, and 1 had vascular infection. Half of the patients had positive IgG aCL titers.

### Peculiarities of Q Fever in French Guiana

In French Guiana, the ratio of men to women was 1.5, and acute pneumonia represented clinical presentation in 154 of 220 (70.0%). Only 13 patients (5.9%) presented with elevated aCL antibodies in the acute phase of the disease, which is much lower than that observed in metropolitan France (1115 of 2105 [53.0%]) (*P* < .001).

### Host Factors

#### Immunocompromised Patients

Ninety-one patients were immunocompromised. Among these, 52 were receiving immunosuppressive therapy. Sixty-six patients (72.5%) had acute Q fever, and 31 (34.1%) presented with persistent focal infection (eTable 5 in the Supplement).

#### Children

Among 58 children, 4 were neonates. Fourteen children presented with persistent endocarditis, whereas lymphadenitis was the unique clinical presentation in 2 children (eTable 12 in the Supplement).

#### Pregnant Women

Thirty-six included patients were pregnant women (mean [SD] age, 30 [6] years). Infection occurred mostly during the 6 first months of pregnancy. Pregnancy complications were identified in 22 of these patients (61.1%) (eTable 6 in the Supplement).

#### Sex

Being male was associated with an increased risk of vascular infection independent of age (odds ratio [OR], 3.4; 95% CI, 2.0-5.7; *P* < .001). Men presented with higher IgG aCL titers than women in the primary phase of the disease (OR, 1.6; 95% CI, 1.3-2.1; *P* < .001), independent of age. Twenty-seven of 58 children (46.6%) were boys; 1654 of 2376 adults (69.6%) were men (*P* < .001).

### Mortality and Complications

**Figure 3.**
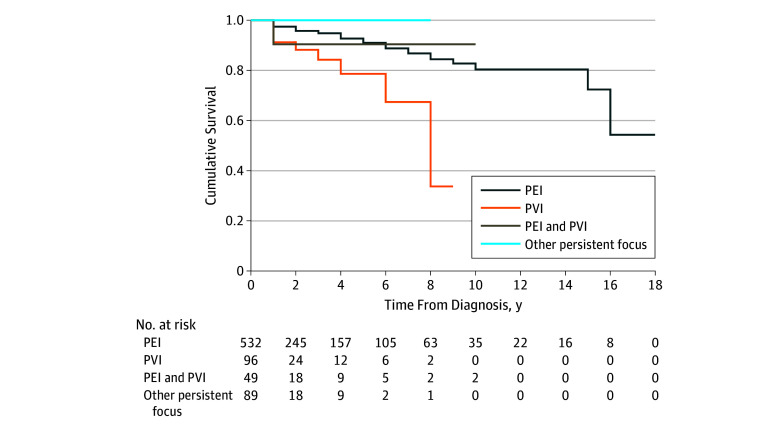
Kaplan-Meier Survival Analysis Includes patients with *Coxiella burnetii* infection. PEI indicates persistent endocarditis; PVI, persistent vascular infection.

Fifty-eight (2.4%) of the 2434 patients diagnosed with Q fever died. Among these, 46 were men (79.3%), the mean (SD) age at the time of Q fever diagnosis was 65.8 (12.9) years, and the mean (SD) age at the time of death was 69 (12) years. Among the 1806 patients with acute Q fever, 3 died (2 of complications of a solid tumor, and 1 of fulminant Q fever hepatitis). Among 766 patients with persistent focalized infection, 55 died, 43 had endocarditis (4 with concomitant vascular infection), and 16 had a vascular infection (of whom 4 had concomitant spondylodiscitis) (Figure 3).

#### Factors Associated With Mortality

Among the 2434 patients with Q fever (accounting for a total duration of follow-up of 3276 person-years, with a median follow-up duration of 4.6 months [interquartile range, 0.1-18.1 months]), the mortality rate was 1.8 (95% CI, 1.4-2.3) per 100 person-years. The mortality rate was 2.4 (95% CI, 1.8-3.3) per 100 person-years among patients with *C burnetii* persistent endocarditis, and 6.2 (95% CI, 3.8-10.1) per 100 person-years among patients with *C burnetii* vascular infection.

All Cox proportional hazards regression risk models used to test the association between each condition and mortality were adjusted for sex, age, and calendar period. Persistent focal *C burnetii* infections were associated with an increased risk of death (hazard ratio [HR], 10.9; 95% CI, 3.2-37.1; *P* < .001). Persistent endocarditis (HR, 2.4; 95% CI, 1.1-5.1; *P* = .02) and vascular infection (HR, 3.1; 95% CI, 1.7-5.7; *P* < .001) were associated with an increased risk of death. Meningitis (HR, 4.0; 95% CI, 1.4-11.6; *P* = .009) and spondylodiscitis (HR, 8.3; 95% CI, 3.3-20.9; *P* < .001), when associated with cardiovascular infection, were also associated with a higher risk of death.

#### Factors Associated With Complications

According to the Cox proportional hazards regression model with lymphoma as the dependent variable, after adjustment for sex, age, and year category at baseline, we found that the presence of lymphadenitis was associated with a higher risk of lymphoma (HR, 77.4; 95% CI, 21.2-281.8; *P* < .001) as was the presence of hemophagocytic syndrome (HR, 19.1; 95% CI, 3.4-108.6; *P* < .001). Valvulopathy, thrombosis, lymphadenitis, maximum high IgG titer (>800), acute Q fever endocarditis, and male sex were identified as factors associated with evolution to persistent focal *C burnetii* infection (Table 1).

IgG anticardiolipin titers were available for 1328 patients (54.6%). Positive aCL antibody findings were indicative of acute Q fever endocarditis (incidence rate ratio, 3.9; 95% CI, 2.0-7.5; *P* < .001) and hemophagocytic syndrome (all patients in this group were positive for aCL antibodies) (Table 2). Immunosuppression was not indicative of any complication. In case of acute Q fever, the receiver operating characteristics analysis showed that the presence of aCL antibodies were significantly associated with acute Q fever complications such as acute Q fever endocarditis (area under the curve [AUC], 0.67; 95% CI, 0.58-0.76; *P* < .001), thrombosis (AUC, 0.72; 95% CI, 0.60-0.85; *P* = .002), hemophagocytic syndrome (AUC, 0.78; 95% CI, 0.67-0.89; *P* = .003), meningitis (AUC, 0.68; 95% CI, 0.56-0.79; *P* = .01), and alithiasic cholecystitis (AUC, 0.75; 95% CI, 0.60-0.90; *P* = .05) (eTable 13 in the Supplement).

## Discussion

We present a comprehensive description of a 26-year cohort of patients with Q fever from the French National Reference Center for Q fever. Endocarditis was the second infectious *C burnetii* focus identified.

New complications identified included acute Q fever endocarditis, hemophagocytic syndrome, thrombosis, lymphadenitis, and lymphoma. Anticardiolipin antibodies (IgG aCL) during acute Q fever were indicative of hepatitis, cholecystitis, endocarditis, thrombosis, hemophagocytic syndrome, meningitis, and progression to persistent endocarditis. Meningitis and spondylodiscitis complications were associated with an increased risk of death.^20^

In the Netherlands, where Q fever has been responsible of 4000 cases in 4 years, physicians used a definitive criterion based on a serologic cutoff rather than the clinical features. Consequently, Q fever complication and unusual presentation of the diseases are probably underestimated.^21,22,23^ In the Netherlands, the mortality rate varies from 1% in cases of acute Q fever to 13% in cases of persistent focalized *C burnetii* infection (9% for endocarditis and 21% for vascular infection).^22,24^ To our knowledge, no study on aCL associated with Q fever has been published from the Netherlands. In addition, TTE is not performed in case of acute Q fever.^25^ Therefore, comparison of the clinical features observed herein with those observed in the Netherlands remains difficult.

By contrast, in French Guiana, clinical manifestations of the disease presented some peculiarities.^26^ Strikingly, the disease affects men and women almost equally. Although the Guiana strain has been described as a highly virulent strain in vivo (unpublished data; C.M., Aurelia Caputo, PhD, Yassina Bechah, PhD, et al; June 2018), no elevation of aCL antibody levels was observed in this region. Regarding lymphoma, a prospective study needs to be performed with the definition criteria used herein.

### Host Factors

In pregnant women, the highest proportion of complications (61.1%) corroborates previous dramatic reports on Q fever during pregnancy.^27^ Placentitis and microthrombi have been described.^23^ In children, Q fever has been marked by an age-related increase in incidence.^28^ The imbalance in the sex ratio distribution of the disease occurred after puberty, and males were most affected by vascular infections and presented with a higher secretion of aCL antibodies during acute Q fever. Thus, sex hormones likely influence the host’s response during Q fever, and further investigation is warranted to determine the mechanism involved.^29^

### New Clinical Manifestations

#### Lymphadenitis and Lymphoma

In 2015, Melenotte et al^13^ described an association between *C burnetii* infection and non–Hodgkin lymphoma. In that study, *C burnetii* lymphadenitis and hemophagocytic syndrome were identified as risk factors of lymphoma. To determine how bone marrow and lymph nodes could influence lymphomagenesis, further investigations are warranted. In any event, because lymphadenitis was the unique infective focus of *C burnetii* in 23 patients (0.9%) in our cohort, and because lymphadenitis was identified in 43.9% of the cases with PET scan imaging, the latter is justified to identify *C burnetii* lymphadenitis as a prelymphomatous stage.^3^

#### Coagulation Disorder and Elevated aCL Levels

##### Acute Q Fever Endocarditis

First described as a subacute disease and later considered a fatal chronic disease, heart valvular injury is now an acute Q fever clinical entity.^30,31^ Acute Q fever endocarditis is associated with evolution to persistent *C burnetii* endocarditis and must be seriously considered with immediate and systematic TTE.^17^

##### Atypical Presentation of Q Fever

The proportion of neurologic involvement (1.3%) in our cohort is consistent with that in 14 previous international publications.^2,32,33,34^ Capillary thrombi and small perivascular hemorrhages have been described, and *C burnetii* has been identified by immunofluorescence in the brain.^35,36,37^ Regarding pericarditis and myocarditis, systematic TTE and the systematic prescription of serologic findings for *C burnetii* have improved the diagnosis of Q fever pericarditis.^38^ Among 22 cases of myocarditis described in the literature, myocardial necrosis has been anecdotally reported.^9,39,40^ Cases of optic neuritis (n = 6) and uveitis (n = 21) reported in the literature have been considered to be inflammatory phenomena triggered by the bacterium without evidence of *C burnetii* in the intraocular specimen.^41,42,43,44,45^ In the literature, 17 cases of *C burnetii* alithiasic cholecystitis were reported. One case was confirmed by positive polymerase chain reaction results for *C burnetii* in the gallbladder, and 2 were associated with antiphospholipid antibodies.^46,47,48,49,50,51,52,53,54,55,56^ Thirteen cases of Q fever hemophagocytic syndrome associated with acute Q fever have been reported in the literature, and an increase in aCL antibodies was reported in only 1 case.^57,58,59,60,61^ *Coxiella burnetii* interstitial lung disease, which was first described after outbreaks in the United States and Russia, is a rare and severe persistent focal *C burnetii* infection with advanced fibrotic lesions and poor clinical outcome.^62,63,64^ First described in 1983 by Janigan and Marrie,^65^ pseudotumors of the lung are rare manifestations of *C burnetii*.^66^ After resection to exclude a tumoral process, histologic findings showed macrophage infiltration with *C burnetii*.^65,66^ Finally, large vessel vasculitis in association with *C burnetii* infection has been described in 5 case reports.^67,68,69,70^ For one of these cases, a high IgG aCL titer was observed.^68^

### Limitations

Some limitations of the study need to be acknowledged. Six hundred fifteen patients with acute Q fever (25.3%) were lost to follow-up because in most cases their clinical course was favorable, and they no longer consulted their referring physician. In addition, cardiovascular *C burnetii* infections were probably overrepresented in this cohort because, as a reference center, we are solicited for severe *C burnetii* infections. Conversely, the mortality rate might be underestimated because of potential loss to follow-up.

## Conclusions

Based on the new definition criteria, hitherto neglected foci of infection include the lymphatic system (ie, bone marrow, lymphadenitis) with a risk of lymphoma. Cardiovascular infections were the main fatal complications, highlighting the importance of routine screening of valvular heart disease and vascular anomalies during acute Q fever. Routine screening for aCL antibodies during acute Q fever can help prevent complications. Further investigations are necessary to evaluate the addition of hydroxychloroquine sulfate to doxycycline in cases of elevated aCL titers. A PET scan could be performed for all patients with suspected persistent focalized infection for early diagnosis of vascular and lymphatic infections associated with death and lymphoma, respectively.

## Notes

Supplement. **eFigure 1.** Serological Test Performed Each Year in the French National Reference Center of *Coxiella burnetii* Infection **eFigure 2.** Standardized Questionnaire for Q Fever Cases in the French National Reference Center **eFigure 3.** Age and Sex Distribution at Diagnosis of *C burnetii* Infection **eFigure 4.** Number of Cases of Persistent Focalized Infection Over Time Regarding the Use of Systematic TTE (2001) and PET Scanning (2009) **eFigure 5.** Focalized Persistent *C burnetii* Lymphadenitis as the Unique Focus of *C burnetii* Persistent Infection **eFigure 6.** Lymphoma and Q Fever **eTable 1.** Diagnostic Criteria of *C burnetii* Persistent Focalized Infection **eTable 2.** Definition Criteria for Patients With *C burnetii* Persistent Infection and Interstitial Lung Diseases (ILD) **eTable 3.** Diagnostic Criteria of *C burnetii* Acute Endocarditis **eTable 4.** Geographic Origin of Serum Sample **eTable 5.** Immunosuppression Characteristics of Patients (n = 91) **eTable 6.** Clinical Manifestation of Q Fever During Pregnancy (n = 36) **eTable 7.** Clinical Presentation of Acute Q Fever in 1806 Patients **eTable 8.** Patients With Q Fever Lymphadenitis (n = 97) **eTable 9.** Clinical Presentation of Persistent *C burnetii* Infections in 766 Patients **eTable 10.** Osteoarticular Infection in Q Fever (n = 56) **eTable 11.** Diagnosis of *C burnetii* Osteoarticular Infection **eTable 12.** *C burnetii* Infection in Children (n = 58) **eTable 13.** ROC Analysis of IgG Anticardiolipin Antibodies and Acute Q Fever Complications
