# Erroneous Statement About Ethical Approval of Study of Clinical Features and Complications of *Coxiella burnetii* Infections

**DOI:** 10.1001/jamanetworkopen.2025.20272

**Published:** 2025-06-03

In the Original Investigation “Clinical Features and Complications of *Coxiella burnetii* Infections From the French National Reference Center for Q Fever,” published on August 24, 2018, in *JAMA Network Open*,^1^ the authors erroneously reported that the study had ethical approval. As Dr Melenotte and Ms Zeitoun-Calvo explain in a Comment,^2^ such approval was not required “per Article L1121-4 of the French Public Health Code.” The erroneous statement has been corrected to state “This study was exempt from ethical review and approval per Article L1121-4 of the French Public Health Code.”
