# Supplementary material for: Clinical Features and Complications of Coxiella burnetii Infections From the French National Reference Center for Q Fever
Source: JAMA Netw Open. 2018 Aug 24;1(4):e181580. doi: 10.1001/jamanetworkopen.2018.1580 (PMC6324270; doi:10.1001/jamanetworkopen.2018.1580)
Supplement: Supplement. — eFigure 1. Serological Test Performed Each Year in the French National Reference Center of Coxiella burnetii Infection eFigure 2. Standardized Questionnaire for Q Fever Cases in the French National Reference Center eFigure 3. Age and Sex Distribution at Diagnosis of C burnetii Infection eFigure 4. Number of Cases of Persistent Focalized Infection Over Time Regarding the Use of Systematic TTE (2001) and PET Scanning (2009) eFigure 5. Focalized Persistent C burnetii Lymphadenitis as the Unique Focus of C burnetii Persistent Infection eFigure 6. Lymphoma and Q Fever eTable 1. Diagnostic Criteria of C burnetii Persistent Focalized Infection eTable 2. Definition Criteria for Patients With C burnetii Persistent Infection and Interstitial Lung Diseases (ILD) eTable 3. Diagnostic Criteria of C burnetii Acute Endocarditis eTable 4. Geographic Origin of Serum Sample eTable 5. Immunosuppression Characteristics of Patients (n = 91) eTable 6. Clinical Manifestation of Q Fever During Pregnancy (n = 36) eTable 7. Clinical Presentation of Acute Q Fever in 1806 Patients eTable 8. Patients With Q Fever Lymphadenitis (n = 97) eTable 9. Clinical Presentation of Persistent C burnetii Infections in 766 Patients eTable 10. Osteoarticular Infection in Q Fever (n = 56) eTable 11. Diagnosis of C burnetii Osteoarticular Infection eTable 12. C burnetii Infection in Children (n = 58) eTable 13. ROC Analysis of IgG Anticardiolipin Antibodies and Acute Q Fever Complications [file jamanetwopen-e181580-s001.pdf]

Supplementary Online Content

Melenotte C, Protopopescu C, Million M, et al. Clinical features and complications of *Coxiella burnetii* infections from the French National Reference Center for Q fever. *JAMA Netw Open.* 2018;1(4):e181580. doi:10.1001/jamanetworkopen.2018.1580

eFigure 1. Serological Test Performed Each Year in the French National Reference Center of *Coxiella burnetii* Infection
eFigure 2. Standardized Questionnaire for Q Fever Cases in the French National Reference Center
eFigure 3. Age and Sex Distribution at Diagnosis of *C burnetii* Infection
eFigure 4. Number of Cases of Persistent Focalized Infection Over Time Regarding the Use of Systematic TTE (2001) and PET Scanning (2009)
eFigure 5. Focalized Persistent *C burnetii* Lymphadenitis as the Unique Focus of *C burnetii* Persistent Infection
eFigure 6. Lymphoma and Q Fever
eTable 1. Diagnostic Criteria of *C burnetii* Persistent Focalized Infection
eTable 2. Definition Criteria for Patients With *C burnetii* Persistent Infection and Interstitial Lung Diseases (ILD)
eTable 3. Diagnostic Criteria of *C burnetii* Acute Endocarditis
eTable 4. Geographic Origin of Serum Sample
eTable 5. Immunosuppression Characteristics of Patients (n = 91)
eTable 6. Clinical Manifestation of Q Fever During Pregnancy (n = 36)
eTable 7. Clinical Presentation of Acute Q Fever in 1806 Patients
eTable 8. Patients With Q Fever Lymphadenitis (n = 97)
eTable 9. Clinical Presentation of Persistent *C burnetii* Infections in 766 Patients
eTable 10. Osteoarticular Infection in Q Fever (n = 56)
eTable 11. Diagnosis of *C burnetii* Osteoarticular Infection
eTable 12. *C burnetii* Infection in Children (n = 58)
eTable 13. ROC Analysis of IgG Anticardiolipin Antibodies and Acute Q Fever Complications

This supplementary material has been provided by the authors to give readers additional information about their work.

eFigure 1. Serological Test Performed Each Year in the French National Reference Center of *Coxiella burnetii* Infection

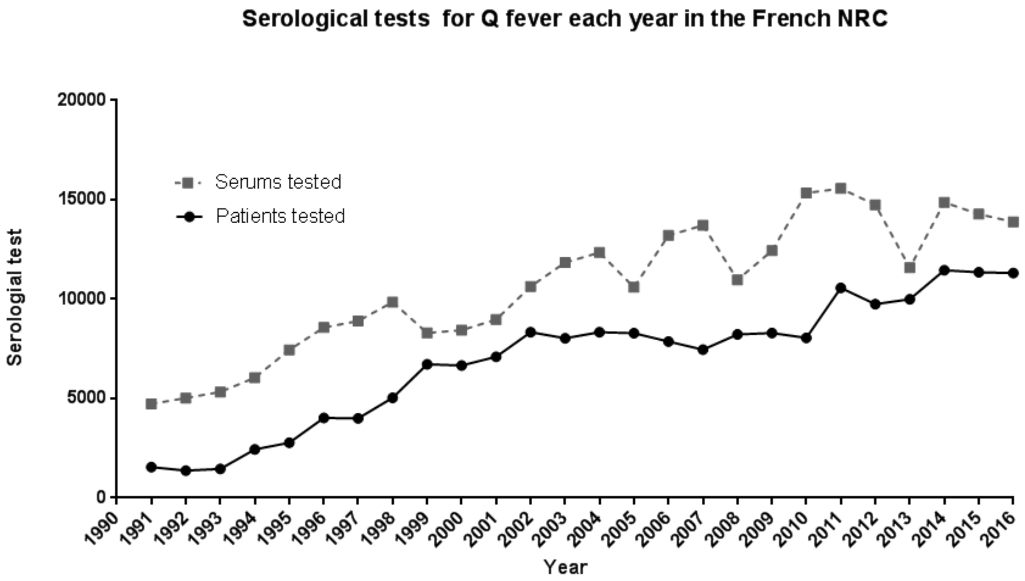

© 2018 Melenotte C et al. *JAMA Network Open.*

eFigure 2. Standardized Questionnaire for Q Fever Cases in the French National Reference

Centre National de Référence des Rickettsies - Pr Didier Raoult

Q Fever

First name<br>Family Name Sex :<br>Date of Birth	Hospital (city and country) :<br>Physician:<br>Tel/Email:

Q fever diagnosis already known:
Transthoracic echography result at the date of the first positive serology in the NRC for Q fever

Acute Q fever Follow up fatigue post acute Q fever
Date of the begining of symptoms
Fever
Acute Q fever on medical predisposition ➔ Valvulopathy
Thrombophlebitis Vascular
Pericarditis Myocarditis Immunosuppression HIV
Other
Neurological form➔ Meningitis
Pneumonia Encephalitis
Acute Hepatitis
Lymphadenitis
Other clinical manifestation

Inflammatory disease
Auto-antibodies
Treatment resistence
Corticoids

Acute Q fever
Acute endocarditis
Follow up of acute Q fever
Possible endocarditis (ABC score)
Certain endocarditis (ABC score)
Possible vascular infection
Certain vascular infection
Lung pseudo tumor
Osteoarticular infection
Other:

CULTURE: Yes No
Cultivation date

Serology

		Phase I			Phase II		
Date	N°	IgG	IgM	IgA	IgG	IgM	IgA

Polymerase chain reaction

Date	Sample	Smarlab	*IS1111*	*IS30*	*Actine*

CULTURE and PCR
Cellular culture
Yes No
MIC =

Second serum request
Yes No

Anticardiolipin antibodies

Treatment
Yes
Antibiotic use:

Date of begning:
Date of the end:

Letter	

According to the Bioethics Act (2004), patients must be informed that the results of investigations may be used for research purposes and published anonymously.

© 2018 Melenotte C et al. *JAMA Network Open.*

eFigure 3. Age and Sex Distribution at Diagnosis of *C burnetii* Infection

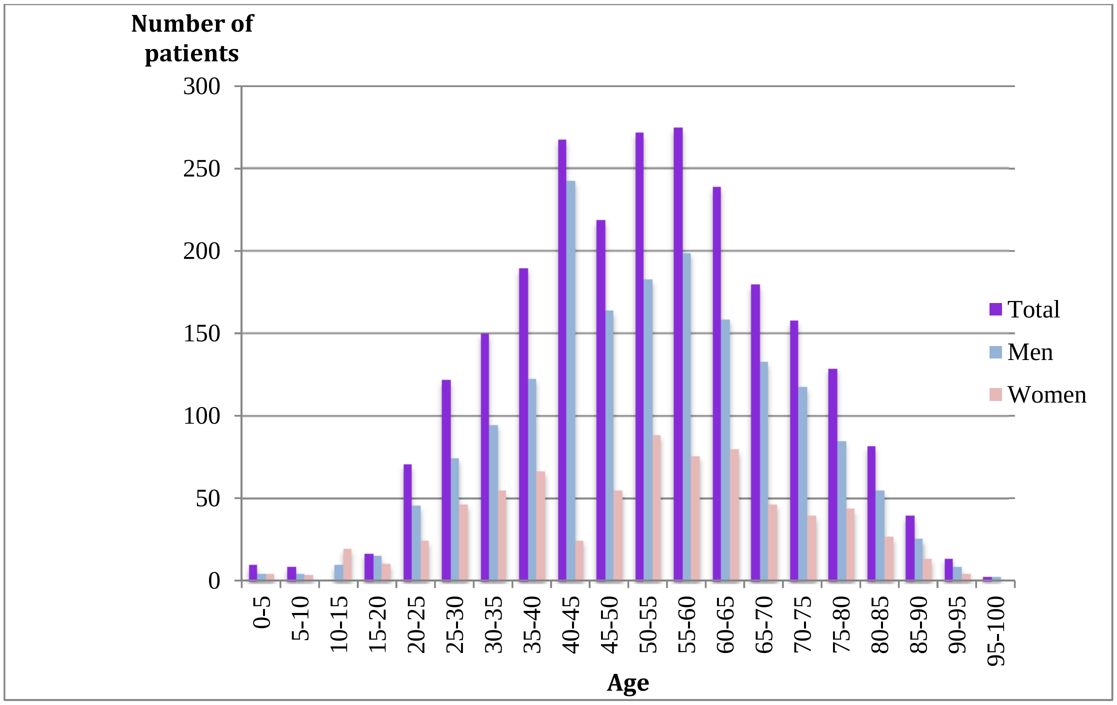

© 2018 Melenotte C et al. *JAMA Network Open.*

eFigure 4. Number of Cases of Persistent Focalized Infection Over Time Regarding the Use of Systematic TTE (2001) and PET Scanning (2009)

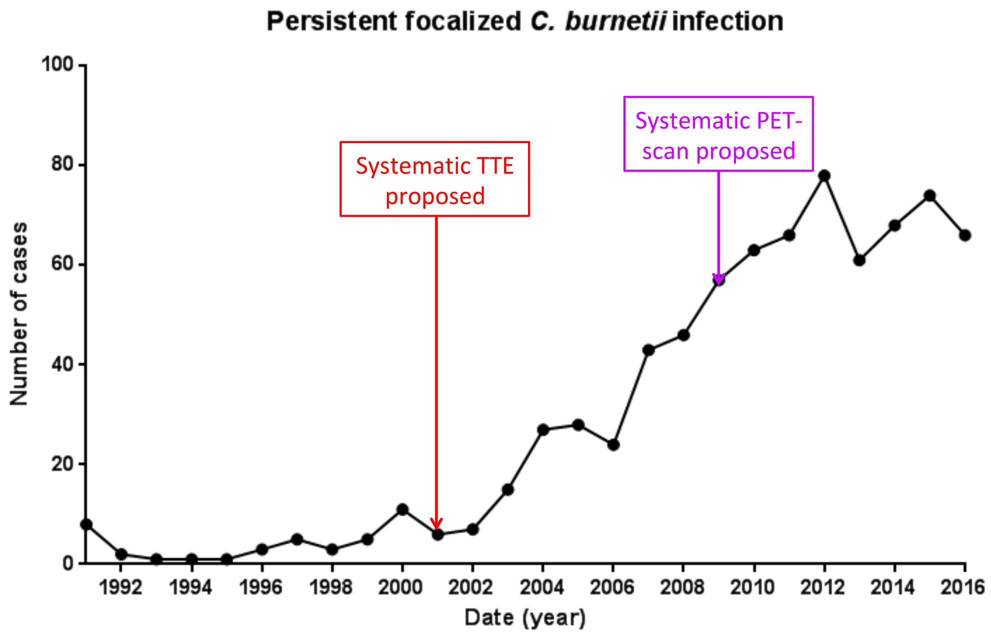

© 2018 Melenotte C et al. *JAMA Network Open.*

eFigure 5. Focalized Persistent *C burnetii* Lymphadenitis as the Unique Focus of *C burnetii* Persistent Infection

Identification of the deep and persistent infective focus with PET-scan.

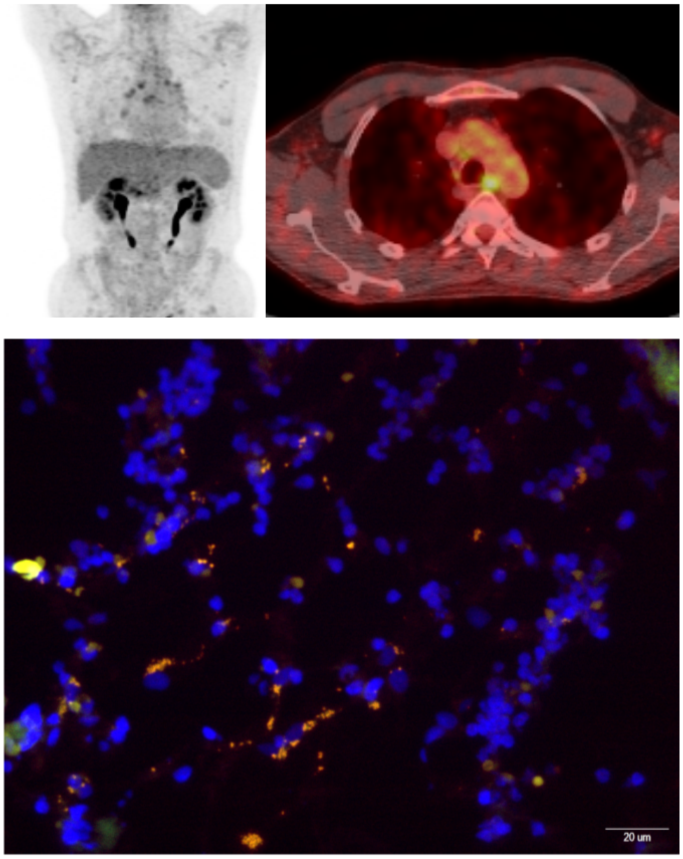

Biopsy revealed positive FISH targeting the cpecific *C. burnetii* 16S rRNA
A & B. PET scan with positive mediastinal lymphadenitis
C. Positive FISH targeting *C. burnetii* 16S rRNA

© 2018 Melenotte C et al. *JAMA Network Open.*

eFigure 6. Lymphoma and Q Fever

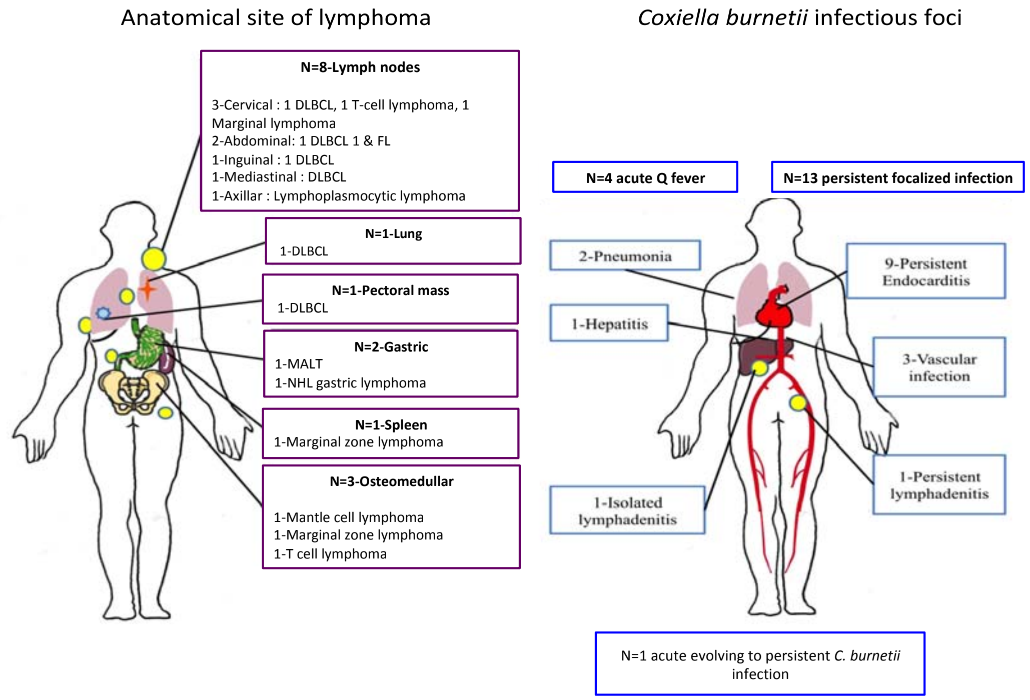

Anatomical foci represent the biopsy sites that led to the diagnosis of lymphoma
The histological type of lymphoma is indicated
DLBCL: Diffuse and large B-cell lymphoma
FL: Follicular lymphoma
MALT: Mucosal-Associated Lymphoid Tissues
LNH: Non-Hodgkin lymphoma (untyped)
Number indicate the number of cases.

© 2018 Melenotte C et al. *JAMA Network Open.*

eTable 1. Diagnostic Criteria of *C burnetii* Persistent Focalized Infection

[1]Reprinted with permission from Eldin C, Mélenotte C, Mediannikov O, et al. From Q fever to Coxiella burnetii infection: a paradigm change. Clin Microbiol Rev. 2017;30(1):115-190.

**C. *burnetii* endocarditis**	***C. burnetii* vascular infection [3]**	***C. burnetii* prosthetic joint arthritis**	***C. burnetii* osteoarticular infection (without prosthesis).**	***C. burnetii* lymphadenitis**
**A. Definite criterion**<br>Positive culture, PCR, or immunochemistry of a cardiac valve	**A. Definite criterion**<br>Positive culture, PCR or immunochemistry of an arterial sample (prosthesis or aneurism) or a periarterial abscess or a spondylodiscitis linked to aorta.	**A. Definite criterion**<br>Positive culture, polymerase chain reaction, or immunochemistry of a periprosthetic biopsy or joint aspirate	**A. Definite criterion**<br>Positive culture, PCR or immunochemistry of bone or synovial biopsy, joint aspirate.	**A. Definite criterion**<br>Positive culture, PCR, immunohistochemistry or fluorescence in situ hybridization of lymphadenitis.
**Major criteria**<br>***Microbiology***:<br>positive culture or PCR of the blood, an emboli or serology with IgG1 antibody titer ≥6400<br>***Evidence of endocardial involvement:***<br>-Echocardiogram positive for IE: oscillating intracardiac mass on valve or supporting structures, in the path of regurgitant jets or on implanted material in the absence of an alternative anatomic explanation; or abscess; or new partial dehiscence of a prosthetic valve; or new valvular regurgitation (worsening or	**Major criteria**<br>***Microbiology***:<br>Positive culture, PCR of the blood or emboli, or serology with IgGI antibodies ≥6400<br>***Evidence of vascular involvement:***<br>-CT-scan: aneurism or vascular prosthesis + periarterial abscess, fistula, or spondylodiscitis.<br>-Pet-san specific fixation on an aneurism or vascular prosthesis.	**Major criteria**<br>***Microbiology:***<br>Positive culture or polymerase chain reaction of the blood<br>-Positive Coxiella burnetii serology with IgGI antibodies ≥ 6400<br>***Evidence of prosthetic involvement:***<br>-Computed tomography scan or MRI positive for prosthetic infection: collection or pseudo-tumor of the prosthesis<br>-Positron emission tomography scan or indium leukocyte scan showing a specific prosthetic hypermetabolism consistent	**B. Major criteria**<br>***Microbiology***:<br>-Positive culture or positive PCR of the blood<br>-Positive serology with IgGI antibodies ≥ 800<br>***Evidence of bone or joint involvement:***<br>-Clinical arthritis, osteitis or tenosynovitis<br>-CT-scan or ultrasonography (for joint) or MRI: osteo-articular destruction, joint effusion, intra-articular collection, spondylodiscitis, synovitis, acromio-clavicular localization.<br>-Pet-scan or indium leukocyte scan showing a specific osteo-articular	**B. Major criteria**<br>***Microbiology***:<br>-Positive culture or positive PCR of the blood<br>-Positive serology with IgGI antibodies ≥ 800<br>***Evidence of lymph node involvement:***<br>-Clinical lymphadenitis<br>-CT-scan or ultrasonography (for joint) or MRI: lymphadenitis > 1cm.<br>-Pet-scan showing specific lymph node uptake.

© 2018 Melenotte C et al. *JAMA Network Open.*

changing of preexisting murmur is not sufficient).<br>-PET scan displaying a specific valve fixation and mycotic aneurism.		with infection†	uptake.	
**Minor criteria**<br>-Predisposing heart condition (known or found on ultrasound)<br>-Fever, temperature > 38°C<br>-Vascular phenomena, major arterial emboli, septic pulmonary infarcts, mycotic aneurysm (observed during PET scan), intracranial hemorrhage, conjunctival hemorrhages and Janeway lesions.<br>-Immunologic phenomena: glomerulonephritis, Osler's nodes, Roth spots, or rheumatoid factor.<br>-Serological evidence: IgG1 antibody titers ≥ 800 < 6400	**Minor criteria**<br>-Serological IgGI ≥ 800 < 6400<br>-Fever, temperature ≥38°C<br>-Emboli<br>-Underlying vascular predisposition (aneurism or vascular prosthesis)	**Minor criteria**<br>-Presence of a joint prosthesis (indispensable criteria)<br>-Fever, temperature >38°C<br>-Joint pain<br>-Serologic evidence: positive C. burnetii serology with IgGI antibodies >800 and <6400 mg/dL	**Minor criteria**<br>-Serological IgGI ≥ 400 < 800 mg/dL<br>-Fever, temperature ≥38°C<br>-Mono- or polyarthralgia	**Minor criteria**<br>-Serological IgGI ≥ 400 < 800mg/dL<br>-Fever, temperature ≥38°C
Definite diagnosis<br>1) 1A criterion<br>2) 2B criteria<br>3) 1B criterion and 3C criteria (including 1 microbiological characteristic and a cardiac predisposition)<br><br>***Possible diagnosis***<br>1) 1B criterion and 2C criteria (including 1	***Definite diagnosis***<br>1) A criterion<br>2) 2B criteria<br>3) 1B criterion and 2C criteria (including 1 microbiological characteristic and a vascular predisposition)<br><br>***Possible diagnosis***<br>Vascular predisposition, serological evidence and fever or emboli.	***Definite diagnosis***<br>1) 1 A criterion<br>2) 2 B criteria<br>3) 1 B criterion and 3 C criteria (including 1 piece of microbiology evidence and presence of a joint prosthesis)<br><br>***Possible diagnosis***<br>1) 1 B criterion, 2 C criteria (including 1 piece of microbiology evidence and presence of a joint	***Definite diagnosis***<br>1 A criterion<br>2 B criteria<br>1B criterion and 3C criteria (including 1 microbiological characteristic)<br><br>***Possible diagnosis***<br>1 B criterion and 2 C criteria<br>3 C criteria	***Definite diagnosis***<br>1 A criterion<br>2 B criteria<br>1B criterion and 2C criteria (including 1 microbiological characteristic)<br><br>***Possible diagnosis***<br>1 B criterion and 1 C criteria<br>2 C criteria

© 2018 Melenotte C et al. *JAMA Network Open.*

microbiological characteristic and a cardiac predisposition)<br>2) 3C criteria (including 1 microbiological characteristic and a "cardiac predisposition)		prosthesis)<br>2) 3 C criteria (including positive serology and presence of a joint prosthesis)		

© 2018 Melenotte C et al. *JAMA Network Open.*

eTable 2. Definition Criteria for Patients With *C burnetii* Persistent Infection and Interstitial Lung Diseases (ILD)

C. burnetii **persistent infection with ILD.**
**A. Definite criteria**<br>Positive culture, PCR, immunochemistry or FISH of lung fibrotic biopsy
**B. Major criteria**<br>Microbiology:<br>1. Positive culture or positive PCR of the blood<br>2. Positive serology with IgGI antibodies ≥ 400<br>Evidence of lung involvement:<br>**AND** thoracic CT-scan imaging of ILD
Diagnosis definite<br>1 A criterion<br>***Possible diagnosis***<br>≥ 2 B criteria (including thoracic CT scan imaging of ILD)

[a] Presence of a reticular pattern, bronchiectasis or honeycombing with basal and peripheral predominance and no condensation, no cysts, no micro nodules, no trapping, no ground glass predominance, which persisted on repeated CT-scan examination.

© 2018 Melenotte C et al. *JAMA Network Open.*

eTable 3. Diagnostic Criteria of *C burnetii* Acute Endocarditis

**Acute *Coxiella burnetii* endocarditis**
**Major criteria** within three months of the onset of symptoms<br>• Microbiological criteria<br>o IgG levels ≥ 200 & IgM levels ≥ 50 for phase II<br>**OR**<br>o Positive PCR on blood sample<br>**OR/AND**<br>o Positive culture on blood sample<br>• Echocardiographic criteria (TTE or TEE)<br>o Valvular vegetation or nodule<br>o Chordae tendineae rupture
**Minor criteria echocardiographic criteria (TTE or TEE) within three months of the onset of symptoms**<br>• Valve thickening<br>• Chordae tendineae thickening<br>• Valve remodeling<br>• Calcification
C. burnetii* acute endocarditis is definite**<br>Two major criteria are fulfilled: one microbiological and one echocardiography criterion<br>C. burnetii* acute endocarditis is possible**<br>One major microbiological criterion & one minor criterion based on echocardiographic results

© 2018 Melenotte C et al. *JAMA Network Open.*

eTable 4. Geographic Origin of Serum Sample

Origin	Number of patients	%
**Metropolitan France**	**2,105**	**86%**
**Europe**	**66**	**2.7%**
Germany	2	0.08%
United kingdom	10	0.4 %
Austria	1	0.004 %
Belgium	6	0.24%
Spain	4	0.16%
Finland	1	0.04%
Hungary	1	0.04%
Ireland	2	0.08%
Italy	27	1.1%
Luxembourg	4	0.16%
Sweden	1	0.04%
Swiss	7	0.28%
**Africa**	**10**	**0.4%**
Algeria	1	0.04%
Benin	1	0.04%
Cameroun	1	0.04%
Senegal	1	0.04%
Tchad	1	0.04%
Reunion Island	5	0.28%
**Middle East**	**17**	**0.69%**
Saudi Arabia	1	0.041%
Egypt	1	0.041%
Israel	16	0.65%
**United States of America**	**9**	**0.36%**
**Latin America**	**222**	**9.1%**
Equator	1	0.04%
Peru	1	0.04%
French Guiana	220	9 %
**Asia**	**5**	**0.2%**
India	1	0.04%
Thailand	4	0.16%

© 2018 Melenotte C et al. *JAMA Network Open.*

eTable 5. Immunosuppression Characteristics of Patients (n = 91)

Immunosuppression	N=91	3.6%
**Immunosuppressive therapy for**		
Auto-immune disease	51	56%
Cancer	6	6.5%
Transplantation	15	16.4%
Renal	10	10.9%
Hepatic	3	3.3%
Cardiac	1	1.1%
Hematopoietic	1	1.1%
**Organ insufficiency**	**5**	**5.5%**
Renal hemodialysis	3	3.3%
Hepatocellular insufficiency	2	2.2%
**Splenectomy**	**10**	**10.9%**
**Viral infection**	4	4.4%
HIV (CD4<200)	2	2.2%
VHC Interferon-Ribavirine	2	2.2%
**Immunosuppressive drugs**	52	57%
Unknown	24	26%
Corticosteroid	28	30.8%
Azathioprine	11	12.1%
Methotrexate	9	10%
Ertanercept (anti TNF-α)	6	6.5%
Mycophenolate mofetil	2	2.2%
Rituximab	2	2.2%
Infliximab	2	2.2%
Salazopyrine	1	1.1%
Thalidomide	1	1.1%
Sirolimus	1	1.1%
Adalimumab	1	1.1%
Acute Q fever	66	72%
Persistent focalized infection	31	34%

© 2018 Melenotte C et al. *JAMA Network Open.*

eTable 6. Clinical Manifestation of Q Fever During Pregnancy (n = 36)

	n=36	1.4%
**Mean age**±SD	30.2±6.4	-
**Term of pregnancy**		
1st trimester	9	25%
2sd trimester	10	27%
3rd trimester	4	11%
NA	13	36%
Valvulopathy	5	13%
**Primary infection**	16	72%
Pneumonia	3	8%
Hepatitis	7	19%
Lymphadenitis	1	2.7%
Thrombosis	1	2.7%
Fever only	4	38%
**Endocarditis**	3	8.3%
**Pregnancy complication**	22	61%
Intrauterine fetal death	5	13.8%
Intrauterine growth retardation	6	16%
Spontaneous abortion	9	25%
Medical termination of pregnancy	2	5.5%

© 2018 Melenotte C et al. *JAMA Network Open.*

eTable 7. Clinical Presentation of Acute Q Fever in 1806 Patients

	**n=1806**	Percentage
Mean age	48.8 ± 16.5 years	-
Follow up duration	11.4 ± 21.8 months	-
Predisposing valvulopahy	255	10.4%
No predisposing valvulopathy	997	40.9%
Unknown predisposing valvulopathy	554	22.7%
Immunosuppression	66	3.6%
Hepatitis	836	46.3%
Pneumonia	480	26.6%
Hepatitis + pneumonia	141	7.8%
Flu like syndrome or isolated fever	350	19.3%
Lymphadenitis	66	3.7%
Lymphadenitis + hepatitis	24	1.3%
Lymphadenitis +pneumonia	16	0.9%
Acute Q fever endocarditis	50	2.8%
Thrombosis	16	0.9%
Pregnancy	16	0.9%
Meningitis and or encephalitis	25	1.4%
Meningoencephalitis	8	0.4%
Meningitis	16	0.6%
Encephalitis	1	0.05%
Alithiasic cholecystitis	11	0.6%
Pericarditis	23	1.3%
Hemophagocytic syndrome	9	0.5%
Myocarditis	7	0.4%
Imprecise	127	7%

© 2018 Melenotte C et al. *JAMA Network Open.*

eTable 8. Patients With Q Fever Lymphadenitis (n = 97)

**Lymphadenitis**	**n=97**	**%**
Mean age (years)	55±19	
Severe immunosuppression	1	1%
Preexisting valvulopathy	24	24%
**Association with other *C. burnetii* focus**		
Hepatitis	30	30%
Pneumonia	18	18%
Pericarditis	2	2%
Meningitis	2	2.1%
Myocarditis	1	1%
Thrombosis	1	1%
Acute Q fever	66	68%
Persistent *C. burnetii* infection	45	46%
Endocarditis	26	27%
Vascular infection	6	6%
Osteoarticular infection	4	4%
Isolated lymphadenitis	23	23%
PET scan contribution to *C. burnetii* lymphadenitis	18/41	44%
Mediastinal	14	14%
Retroperitoneal	1	1%
Sus clavicular	3	3%
Inguinal	3	3%
Cervical	3	3%

© 2018 Melenotte C et al. *JAMA Network Open.*

eTable 9. Clinical Presentation of Persistent *C burnetii* Infections in 766 Patients

	Endocarditis N=581		Vascular infection N=145		Osteo articular infection N=56	
Age (mean±SD)	59.4±17.3	-	63.4±14.3	-	59.6±19.9	-
Sex (men)	419	72.1%	127	88.2%	37	66.1%
Immunosuppression	22	3.8%	6	4.2%	1	1.8%
Valvular predisposition	449	77.4%	57	39.6%	7	12.5%
Prosthetic material	204	35%	62	44%	10	17.8%
Endocarditis	-	-	49	34.0%	7	12.5%
Vascular infection	49	8.4%	-	-	11	19.2%
Osteoarticular infection	8	1.3%	11	7.5%	-	-
Hepatitis	123	21.2%	28	19.4%	6	10.7%
Pneumonia	52	8.9%	10	6.9%	2	3.6%
Lymphadenitis	26	4.5%	6	4.2%	4	7.1%
Acute endocarditis	13	2.2%	1	0.7%	0	0%
Lymphoma	10	1.7%	2	1.4%	0	0%
Meningitis	7	1.2%	0	0%	1	1.8%
Hemophagocytic syndrome	1	0.2%	1	0.7%	0	0%

© 2018 Melenotte C et al. *JAMA Network Open.*

eTable 10. Osteoarticular Infection in Q Fever (n = 56)

**Osteo articular infection**	**N=56**	**%**
Mean age	59.6±19.9	-
Sex	37	66.1%
Immunosuppression	1	1.8%
**Site of osteo articular infection**		
Spondyliscitis	24	23%
Acromio-clavicular	4	7%
Knee	9	16%
Hip	6	10.7%
Other	3	5.3%
Tibial osteomyelitis	1	1.7%
Cuneiform bone feet	1	1.7%
Tenosynovite feet	1	1.7%
Bursite shoulder	1	1.7%
Sternum	1	1.7%
Humerus	1	1.7%
Tenosynovite des flechisseurs de la main	1	1.7%
Ankle	3	5.3%
**Associated focus**		
Persistent cardio vascular infection	17	30.3%
Vascular infection	11	19.6%
Persistent endocarditis	8	14%
Vascular infection+ Persistent endocarditis	2	3.6%
Lymphadenitis	4	7.4%

© 2018 Melenotte C et al. *JAMA Network Open.*

eTable 11. Diagnosis of *C burnetii* Osteoarticular Infection

**Diagnostis test in addition to the positive serology**	**N=27/56**	**48%**
Positive culture	3	5.3%
Positive PCR	26	46%
Bone	17	30%
Joint Fluid	3	5.3%
Blood	2	3.5%
Abcess (Para vertebral/psoas)	5	8.9%)

© 2018 Melenotte C et al. *JAMA Network Open.*

eTable 12. *C burnetii* Infection in Children (n = 58)

	**Patients (n=58)**	**100%**
Age (Mean ±SD)	10±5	-
Sex (boy)	27	46%
**Medical history**	**1**	**1.7%**
Severe immunodeficiency	14	25%
Pre-existing valvulopathy	14	25%
Endocarditis	7	12%
Native valve	7	12%
Prosthetic valve	3	5.1%
**Vascular infection**	**2**	**3.4%**
Prosthetic material	1	1.7%
Native vessel	3	5.1%
Osteomyelitis	22	38%
Isolated Fever	10	17%
Hepatitis	5	8.6%
Pneumonia	3	5.1%
Lymphadenitis	42	72%
Primary *C. burnetii* infection	19	32%
Persistent *C. burnetii* infection	30	51.7%

© 2018 Melenotte C et al. *JAMA Network Open.*

eTable 13. ROC Analysis of IgG Anticardiolipin Antibodies and Acute Q Fever Complications

Variable	AUC	95%CI		*P*
Acute Q fever endocarditis	.67	.58	.76	.0001
Hemophagocytic syndrome	.78	.67	.89	.003
Meningitis	.68	.56	.79	.01
Thrombosis	.72	.6	.85	.002
Alithiasic cholecystitis	.75	.6	.9	.05

AUC: area under curve, CI: confidence interval

© 2018 Melenotte C et al. *JAMA Network Open.*
